# Prenatal Clinical Presentation and Genetic Analysis of Partial Trisomy 12: A Case Report

**DOI:** 10.7759/cureus.67410

**Published:** 2024-08-21

**Authors:** Nnenna Mbara, Adegbenro O Fakoya

**Affiliations:** 1 Obstetrics and Gynaecology, University of Medicine and Health Sciences, Basseterre, KNA; 2 Cellular Biology and Anatomy, Louisiana State University Health Sciences Center, Shreveport, USA

**Keywords:** amniocentesis, mosaicism, genetic analysis, prenatal diagnosis, chromosomal abnormality, trisomy 12

## Abstract

Trisomy 12 is a rare chromosomal abnormality characterized by the presence of an extra copy of chromosome 12 in some or all cells. This condition can present with a variety of phenotypic manifestations, depending on the extent of mosaicism and the specific genes involved. Prenatal diagnosis of trisomy 12 is challenging due to its variable presentation and potential overlap with other chromosomal abnormalities. This case report describes a 23-year-old woman referred to a Maternal-Fetal Medicine (MFM) clinic following abnormal findings on a routine prenatal ultrasound at 20 weeks' gestation. The ultrasound indicated increased nuchal fold, a pleural effusion, clenched hands, shortened long bones, flat facial features, and clubbed feet. Amniocentesis showed a partial trisomy of distal 12q on the cytogenetic band 12q21.2 to 12q24.33. The patient was advised on the need for close monitoring of fetal growth and development through serial ultrasounds and follow-up visits, with a multidisciplinary approach including genetic counseling. This case underscores the importance of comprehensive prenatal ultrasound screening, prenatal genetic diagnosis, and multidisciplinary management in addressing the varied phenotypic manifestations of trisomy 12.

## Introduction

Trisomy 12, a chromosomal anomaly characterized by an extra copy of chromosome 12, manifests in various clinical contexts, most notably in certain hematological malignancies, such as leukemia and congenital abnormalities. Patients with trisomy 12 have an elevated malignancy risk compared to patients without trisomy 12. This genetic aberration leads to changes in cellular apoptosis and contributes to diverse phenotypic outcomes, including developmental delays, distinctive facial features, and a spectrum of organ system involvements [1]. Patients may present with micrognathia, cleft lip and/or palate, hypertelorism, ventriculomegaly, and corpus callosum agenesis. In the realm of oncology, trisomy 12 is frequently associated with chronic lymphocytic leukemia (CLL) and other lymphoproliferative disorders, influencing disease prognosis and therapeutic responses [2]. The underlying molecular mechanisms remain an active area of research, with recent studies highlighting the roles of gene dosage effects, disrupted signaling pathways, and epigenetic modifications [2]. Pregnancies with trisomy 12 are often associated with a high risk of miscarriage and may result in early termination due to severe developmental anomalies. Babies born with trisomy 12 may face significant intellectual disabilities and physical malformations, affecting multiple organs [3]. 

Partial trisomy 12 is associated with a higher risk of intrauterine death, particularly if significant structural abnormalities are present. The prognosis often depends on the specific genes involved in the duplication. If the baby survives to birth, careful postnatal follow-up is essential. Infants with partial trisomy 12 may present with congenital anomalies, developmental delays, and other health issues [3]. The severity can vary widely, but ongoing medical care is often required. The prevalence of partial trisomy 12 is estimated to be very rare, approximately 1 in 10,000 to 1 in 25,000 live births [4]. Genetic testing for partial trisomy 12 is crucial for diagnosing the condition, understanding its extent, and planning appropriate management.

Karyotyping is a traditional method used to visualize chromosomes under a microscope, allowing for the detection of chromosomal abnormalities. This process involves culturing cells, staining the chromosomes, and analyzing them for any extra chromosomal material. For example, in cases of partial trisomy 12, an additional segment of chromosome 12 would be identified using this technique [5]. Fluorescence in situ hybridization (FISH) is another method that detects and localizes specific DNA sequences on chromosomes using fluorescent probes. These probes bind to specific regions of chromosome 12, making it possible to identify extra chromosomal material. FISH is more sensitive than karyotyping, allowing for the detection of smaller duplications [5]. Next-generation sequencing (NGS) offers even more detailed information about a person’s genetic makeup, including the detection of partial trisomy. By sequencing the entire genome or specific regions, NGS allows for the precise identification of duplications with high sensitivity and specificity, making it possible to detect even small duplications on chromosome 12 [5]. In prenatal diagnostics, amniocentesis involves extracting amniotic fluid to analyze fetal DNA for chromosomal abnormalities, while chorionic villus sampling (CVS) samples placental tissue to detect such abnormalities, including partial trisomy 12. Non-invasive prenatal testing (NIPT) is a screening method that uses maternal blood to detect fetal chromosomal abnormalities by analyzing cell-free fetal DNA (cffDNA). Each of these diagnostic tests offers varying levels of detail and specificity, and they are often used in combination to confirm a diagnosis [6].

The diagnosis of trisomy 12 is typically made through chromosomal analysis, such as amniocentesis or CVS, often following abnormal findings on ultrasound or NIPT [6]. Ultrasound markers that may prompt further investigation include increased nuchal fold, pericardial effusion, growth restriction, and other structural anomalies [7]. In this case, the patient's 20-week ultrasound revealed increased nuchal fold and pleural effusion, raising suspicion of a chromosomal abnormality and leading to a referral to the Maternal-Fetal Medicine (MFM) clinic.

## Case presentation

A 23-year-old G2P1 white woman presented to the MFM clinic in Augusta, Georgia, for a referral following an abnormal finding on a routine prenatal ultrasound. Her regular obstetrician-gynecologist (OB-GYN) provider followed the patient at the beginning of the pregnancy with no significant events. She was 22 weeks gestation at the presentation. Her obstetric history included one previous uncomplicated full-term vaginal delivery four years ago. Nothing in her prenatal history, medication history, or family history indicated any concerns with her current pregnancy. Her current pregnancy had been progressing uneventfully until her 20-week anatomy scan revealed concerns.

**Figure 1:**
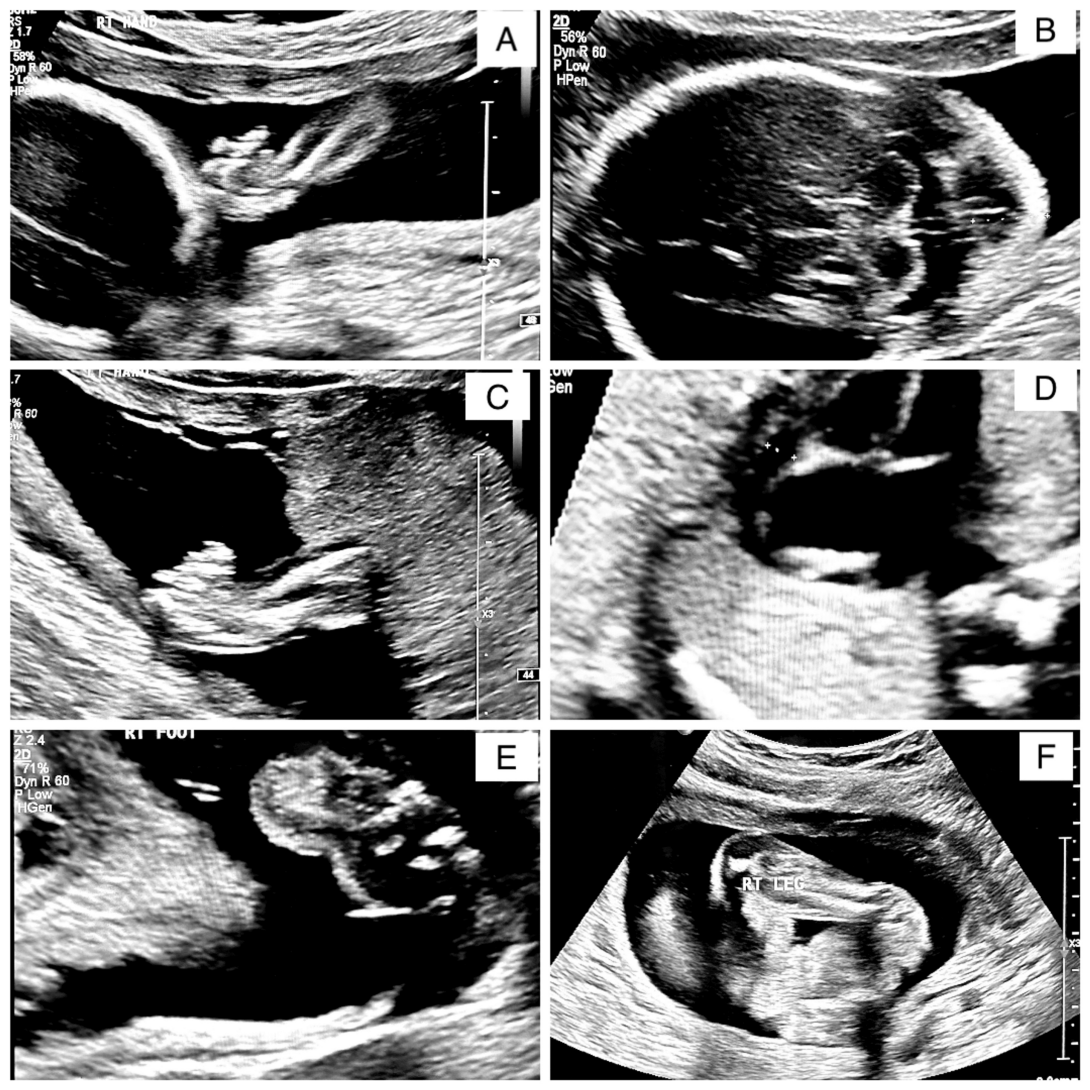
Ultrasound scan at 20 weeks gestation (A) The right upper extremity appears to be shortened and the foot angle appears to be abnormal; (B) Thickened nuchal fold that was measured to be 11.94 mm; (C) The left hand is shown, appears to be in a clenched position; (D) Four chamber of the heart view. A pericardial effusion with an abnormal heart axis; (E) The right foot angle presents in an abnormal fashion suggesting club foot; (F) The right lower extremity appears to be shortened.

The referral report from her primary OB-GYN illustrated a fetus with a fetal heart rate (FHR) of 159 bpm. Estimated fetal weight (EFW) was 265 g (0 lbs 10 oz). The right upper and lower leg and foot angles were abnormal (Figure 1A). There was a thickened nuchal fold (Figure 1B), a questionable flattened nose with frontal facial swelling, and two vessel cords noted. The upper extremities were shortened with bilateral clenched hands (Figure 1C). Additionally, there was a pericardial effusion present, and the heart axis appeared abnormal (Figure 1D). The right foot angle is abnormal, suggesting club foot (Figure 1E), and the right lower extremity appears to be shortened (Figure 1F).

There was a normal amniotic fluid index. The placenta was anterior, with central cord insertion. The cervix was 3.6 cm long and closed. The maternal adnexa was unremarkable. The patient denied any significant medical history, chronic conditions, or current medications, aside from prenatal vitamins. She reported no known genetic conditions in her family and no history of miscarriages or complications in her previous pregnancy.

On examination, the patient appeared well and in no acute distress. Her vital signs were within normal limits. The fundal height was consistent with gestational age, and the FHR was present with a regular rhythm. The MFM specialist recommended a comprehensive fetal echocardiogram and a further detailed ultrasound to assess the fetal heart and other anatomical structures. Additionally, the patient was counseled on the option of NIPT and amniocentesis to rule out chromosomal abnormalities, such as trisomy 21, 18, and 13. The patient opted not to undergo any more genetic testing and chose to proceed with her pregnancy.

An amniocentesis was performed at 22 weeks gestation. A prenatal whole genome chromosomal microarray showed the result of partial trisomy of distal 12q cytogenetic band 12q21.2 to 12q24.33, with female gender. The patient was advised on the potential need for close fetal growth and development monitoring through serial ultrasounds and follow-up visits. The patient was offered a parental karyotype; however, she decided not to undergo any more testing. A multidisciplinary approach, including consultations with a genetic counselor, is suggested to provide the patient with comprehensive care and support throughout her pregnancy. The patient received counseling on pregnancy termination but decided to continue with her pregnancy. At a follow-up appointment at 29 weeks gestation, an ultrasound revealed fetal demise. She subsequently delivered vaginally in the hospital.

## Discussion

Partial trisomy 12 is a rare chromosomal disorder in which only a portion of chromosome 12 is present in triplicate. Understanding the risk factors associated with this condition is crucial for early detection. Some risk factors include advanced maternal age, parental chromosomal abnormalities, and a positive family history of chromosomal abnormalities or a previous pregnancy with chromosomal abnormalities [8]. This chromosomal abnormality leads to a variety of prenatal and postnatal anomalies, contributing to a spectrum of clinical presentations.

Prenatal anomalies associated with partial trisomy 12 are often detected through routine ultrasound screenings and genetic testing during pregnancy. Typical findings include growth retardation. Intrauterine growth restriction (IUGR) is frequently observed, leading to lower birth weights and smaller-than-expected gestational age measurements [8]. Craniofacial anomalies on ultrasounds may reveal characteristic facial dysmorphisms, such as micrognathia, cleft lip and/or palate, and hypertelorism. Congenital heart defects, such as ventricular septal defects (VSDs) and atrial septal defects (ASDs), are commonly detected prenatally. Brain anomalies, including ventriculomegaly and agenesis of the corpus callosum, can be identified during prenatal scans. Limb abnormalities, such as polydactyly and clubfoot, may be present [9]. Polyhydramnios or oligohydramnios may be observed, indicating possible complications.

Following birth, individuals with partial trisomy 12 may present with various anomalies varying in severity. Continued postnatal growth retardation and significant delays in reaching developmental milestones are common. Early intervention programs are crucial to support development [10]. The craniofacial anomalies identified prenatally often persist, and additional features, such as a broad nasal bridge, epicanthal folds, and low-set ears, may be noted. Individuals may experience seizures, hypotonia, and intellectual disabilities of varying degrees [11]. Ongoing neurological assessments and interventions are essential. Feeding difficulties, gastroesophageal reflux, and other gastrointestinal issues may be prevalent. Postnatal assessment often confirms limb abnormalities and may identify additional skeletal anomalies requiring orthopedic management [12]. Due to craniofacial and neurological problems, respiratory complications, such as apnea and recurrent infections, may occur [13].

Partial trisomy 12 requires a multidisciplinary approach involving pediatricians, cardiologists, neurologists, geneticists, and other specialists. Early intervention programs, tailored educational plans, and regular monitoring of growth and development are critical to improving the quality of life for these individuals [14]. The prognosis for individuals with partial trisomy 12 varies widely, depending on the specific genetic abnormalities and the severity of associated anomalies. While some individuals may lead relatively independent lives with appropriate support, others may require lifelong care and medical intervention [14].

## Conclusions

Trisomy 12, while rare, presents significant clinical challenges. Identifying and understanding the risk factors associated with this condition can aid in early detection and intervention. Advanced maternal age, parental chromosomal abnormalities, family history, environmental factors, assisted reproductive technologies, maternal health conditions, and paternal age are all important considerations. Through comprehensive prenatal screening, diagnostic testing, and genetic counseling, healthcare providers can better manage and support affected families. This case report highlights the clinical and genetic complexity associated with trisomy 12, emphasizing its significant role in both prenatal and postnatal contexts. The patient's presentation and subsequent diagnostic evaluations underscore the importance of comprehensive genetic screening and multidisciplinary management in addressing the varied phenotypic manifestations of this chromosomal anomaly. As our understanding of the molecular mechanisms underlying trisomy 12 continues to evolve, it paves the way for more targeted and personalized medical interventions. This case reinforces the necessity for ongoing research and advanced diagnostic techniques to enhance patient care and outcomes in individuals affected by trisomy 12.
